# Shoot gravitropism and organ straightening cooperate to arrive at a mechanically favorable shape in *Arabidopsis*

**DOI:** 10.1038/s41598-023-38069-x

**Published:** 2023-07-17

**Authors:** Satoru Tsugawa, Yuzuki Miyake, Keishi Okamoto, Masatsugu Toyota, Hiroki Yagi, Miyo Terao Morita, Ikuko Hara-Nishimura, Taku Demura, Haruko Ueda

**Affiliations:** 1 Department of Mechanical Engineering, Faculty of Systems Science and Technology, Akita Prefectural University, Yurihonjo, Akita, 015-0055 Japan; 2 Graduate School of Natural Science, Konan University, Kobe, Hyogo, 658-8501 Japan; 3 Graduate School of Science, Kyoto University, Kyoto, 606-8502 Japan; 4 Present Address: Hirano Senior High School Attached to Osaka Kyoiku University, Osaka, 547-0032 Japan; 5 Department of Biochemistry and Molecular Biology, Saitama University, Saitama, 338-8570 Japan; 6 Suntory Rising Stars Encouragement Program in Life Sciences (SunRiSE), Suntory Foundation for Life Sciences, Kyoto, 619-0284 Japan; 7 Department of Botany, University of Wisconsin, Madison, WI, 53706 USA; 8 Division of Plant Environmental Responses, National Institute for Basic Biology, Okazaki, 444-8585 Japan; 9 Course for Basic Biology, The Graduate University for Advanced Studies (SOKENDAI), Hayama, Kanagawa, 240-0115 Japan; 10 Center for Digital Green-innovation, Nara Institute of Science and Technology, Ikoma, Nara, 630-0192 Japan; 11 Graduate School of Science and Technology, Nara Institute of Science and Technology, Ikoma, Nara, 630-0192 Japan; 12 Department of Biology, Faculty of Science and Engineering, Konan University, Kobe, Hyogo, 658-8501 Japan

**Keywords:** Computational biophysics, Mathematics and computing, Tropism

## Abstract

Gravitropism is the plant organ bending in response to gravity, while a straightening mechanism prevents bending beyond the gravitropic set-point angle. The promotion and prevention of bending occur simultaneously around the inflorescence stem tip. How these two opposing forces work together and what part of the stem they affect are unknown. To understand the mechanical forces involved, we rotated wild type and organ-straightening-deficient mutant (*myosin xif xik*) *Arabidopsis* plants to a horizontal position to initiate bending. The mutant stems started to bend before the wild-type stems, which led us to hypothesize that the force preventing bending was weaker in mutant. We modeled the wild-type and mutant stems as elastic rods, and evaluated two parameters: an organ-angle-dependent gravitropic-responsive parameter (*β*) and an organ-curvature-dependent proprioceptive-responsive parameter (*γ*). Our model showed that these two parameters were lower in mutant than in wild type, implying that, unexpectedly, both promotion and prevention of bending are weak in mutant. Subsequently, finite element method simulations revealed that the compressive stress in the middle of the stem was significantly lower in wild type than in mutant. The results of this study show that myosin-XIk-and-XIf-dependent organ straightening adjusts the stress distribution to achieve a mechanically favorable shape.

## Introduction

In tropic response, plant organs undergo differential growth in response to environmental stimuli such as gravity and light. In gravitropism, roots bend down due to gravity and shoots bend up against gravity^1,2^. In this process, endodermal cells in shoots and columella cells in roots sense the direction of gravity by the sedimentation of amyloplasts, which function as statoliths^3–5^. More precisely, experiments have revealed that specific cells in coleoptiles sense the inclination angle of the organs independent of the intensity of gravity, suggesting that the gravity sensor detects the inclination angle of the organ and not the force^6^. Sensing the inclination angle induces a signal transduction pathway that leads to the formation of an auxin gradient and subsequently to differential cell growth. In addition to this gravitational response mechanism, plants have mechanisms that help them maintain a straight posture, possibly by sensing their current curvatures, a phenomenon known as autotropism, autostraightening, or organ straightening^7–10^. This indicates that bending depends on the inclination angle and curvature of the organs involved^11^.

*Arabidopsis thaliana* mutants that are defective in two myosin XI members (myosin XIf and myosin XIk) or ACTIN8 exhibit a hyperbending phenotype in various elongating organs, including inflorescence stems, in response to environmental stimuli, such as gravity and light^9^. To examine organ straightening, clinorotation can be used to neutralize Earth’s unilateral gravitational pull. The wild-type stems straightened their bent shape during clinorotation, but the mutant stems of *myosin xif xik* and *actin8* continued to bend, indicating that these mutants have defects in their straightening ability^9^.Although the actin–myosin XI cytoskeleton is an essential component of the molecular machinery for posture control, their specific function remains unclear.

Mathematical models of straightening are useful to *in-plant* experiments^10–14^. Interestingly, the straightening effect of the stem was theoretically predicted by considering the passive orientation drift of the apical angle, where the angle of the tip changes and drifts indefinitely until growth stops once the stem gains incremental length while maintaining the same curvature^10^. In an early mathematical model, stem curvature was determined using only the inclination angle of the stem segment relative to the ground (gravitropic sensing); however, it was predicted to exhibit oscillatory behavior without posture control. To solve this problem, the previous research proposed a mathematical model that includes curvature sensing of the stem segment (straightening or proprioception) in addition to gravitropic sensing, enabling the stem to control its straightened posture^11^.

Despite these advances in experimental science and mathematical modeling, the spatio-temporal characteristics of stem bending, and subsequent straightening are still not well understood. To quantify these characteristics, we compared bending behavior in the wild type with hyperbending behavior due to defective straightening in *myosin xif xik* mutants. First, we analyzed the morphological differences between wild type and *myosin xif xik* during stem bending in response to gravity. Next, we combined these data with the model to extract the mechanical forces acting during the bending. Finally, we tested the hypothesis that the straightened posture in the wild type is mechanically beneficial with an adjusted stress distribution. Our results indicate that the straightening behavior during bending is mechanically important for plant stems.

## Results

### Quantitative analysis reveals spatio-temporal characteristics of hyperbending stems

**Figure 1:**
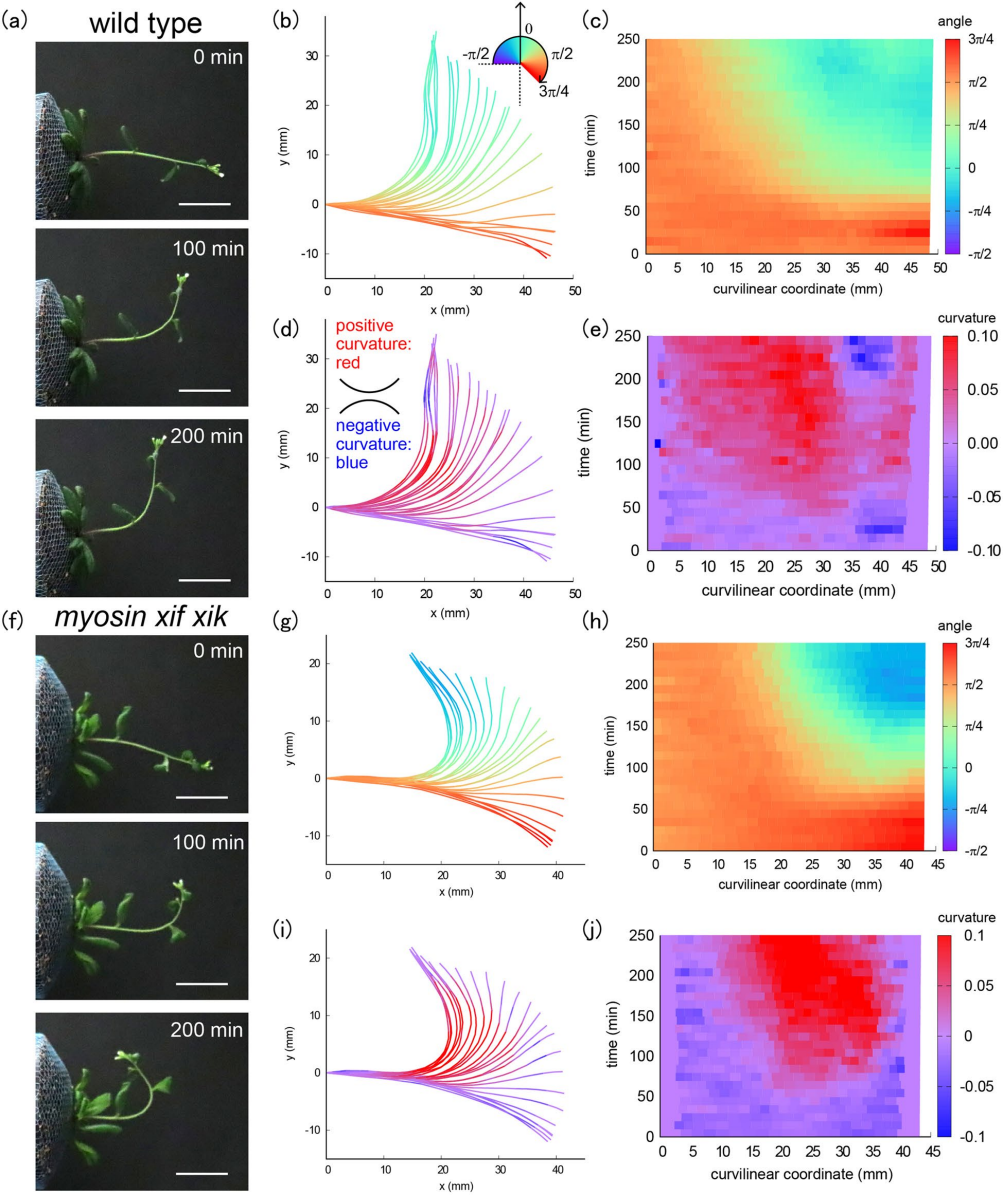
Image analysis of shoot gravitropism over time. (**a**,**f**) Time-lapse images of shoot gravitropism for wild type (t = 0, 100, 200 min) (**a**) and for *myosin xif xik* (**f**). Scale bars 20 mm. (**b**,**c**,**g**,**h**) Color diagrams of the inclination angle of the stem as a function of time (min) and curvilinear coordinates (mm) for wild type (**b**,**c**) and for *myosin xif xik* (**g**,**h**). (**d**,**e**,**i**,**j**) Color diagrams of the curvature of the stem as a function of time (min) and curvilinear coordinate (mm) for wild type (**d**,**e**) and for *myosin xif xik* (**i**,**j**).

To capture the spatio-temporal characteristics of inflorescence stems during bending, we took time-lapse images of wild-type stems and *myosin xif xik* stems (Fig. 1a,f, Figs. S1, S2). We used ImageJ to extract the centerline of each stem from the images and constructed a continuous curve using spline interpolation. The spatio-temporal changes in length and curvature were evaluated for nine stems of wild type and eight of *myosin xif xik*. Representative results of inclination angles and curvatures for each genotype are shown in Fig. 1b–e and g–j, respectively (see Figs. S1, S2 for all examples). The curvilinear coordinate (mm) was defined as the distance along the stem. The inclination angles of the stems changed to positive values (greenish colors) after 70–80 min of gravistimulation for wild type and *myosin xif xik* (Fig. 1b,c,g,h). The stem angle for *myosin xif xik* became negative after 140–150 min (Fig. 1g,h), whereas that of the wild type did not (Fig. 1b,c), indicating that hyperbending behavior occurs in *myosin xif xik* but not in the wild type. Among the nine wild-type stems analyzed, one became negative after 110 min and then returned to positive after 170 min (Fig. S1), showing that wild-type plants also have the potential to overshoot transiently. Overshooting is a well-established phenomenon that occurs in various wild-type plants^15,16^. In contrast, *myosin xif xik*-stems became negative in all eight individual cases, and the negative state was maintained for at least 240 min (Fig. S2). The stem curvature changed to a positive value (reddish colors) after 70–110 min for both genotypes, and the curvature at the tip region (5–10 mm from the tip) remained positive for *myosin xif xik* after 140–170 min, but not for the wild type (Fig. 1d,e,i,j).

**Figure 2:**
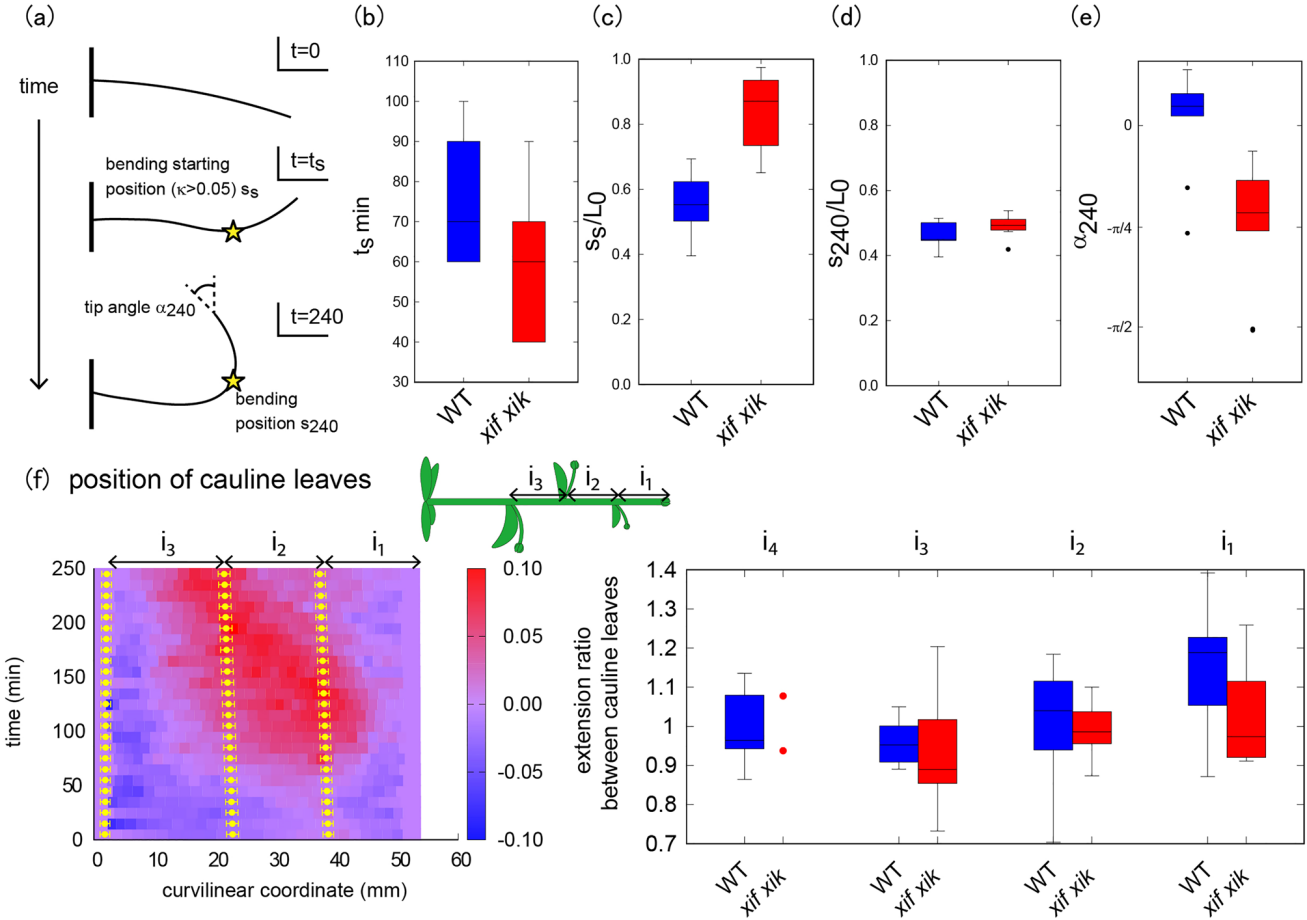
Quantification of stem bending during gravitropic response. (**a**) Schematic illustrations of spatio-temporal characteristics of stem bending. (**b**) Bending starting time ${t}_{s}$. (**c**) Relative bending starting position. (**d**) Relative bending position after 240 min. (**e**) Tip angle (radian) after 240 min. (**f**) Color diagram of the curvature of the stem as a function of time (min) and curvilinear coordinates (mm) for wild type with the position of cauline leaves (left). Extension ratio between cauline leaves (right).

To quantitatively analyze when and where the stem bends and straightens, we defined a few characteristics to capture spatio-temporal behavior during bending (Fig. 2a). The time required to begin gravitropic bending was defined as ${t}_{s}$ when the curvature reached a certain threshold, ${\kappa }_{c}$ ($\kappa >{\kappa }_{c}$, ${\kappa }_{c}=0.05$). When subjected to gravistimulation, the time ${t}_{s}$ for *myosin xif xik* was lower than that for the wild type, indicating that the *myosin xif xik* stems bent earlier than the wild-type stems (Fig. 2b).

Next, we attempted to capture the spatial information of the stems. The initial condition of the inflorescence stem at $t=0$ was approximately horizontal. The starting position of the bending was characterized by position $s$ with a curvature larger than the threshold ${\kappa }_{c}$, denoted as ${s}_{s}$. The value of ${\kappa }_{c}=0.05$ was approximately equal to half the maximum curvature in space and time. To compare individual samples of different lengths, we used the relative position ${s}_{s}/{L}_{0}$ where ${L}_{0}$ is the initial length of the stem. We noted that the total length at time $t$ did not change; therefore, we used the initial length as the representative stem length. The bending position after 240 min was denoted by ${s}_{240}$ and its relative value ${s}_{240}/{L}_{0}$ captured the location of the bending position at that time. The relative starting position ${s}_{s}/{L}_{0}$ of the *myosin xif xik* stem was closer to the tip than that of the wild-type stem, but the relative bending position after 240 min, ${s}_{240}/{L}_{0}$, was almost the same (Fig. 2c,d), suggesting that the elongating region in the *myosin xif xik* stem was different from that in the wild-type stem.

To evaluate the degree of stem bending, we defined the inclination angle after 240 min as ${\alpha }_{240}$ which quantified the direction of the stem tip. The angle ${\alpha }_{240}$ for *myosin xif xik* showed negative values, while the angle ${\alpha }_{240}$ for the wild type was nearly zero (Fig. 2e), supporting the previously reported hyperbending nature of *myosin xif xik* stems^9^. Next, we examined whether axial stem growth affected these characteristics. Elongation of the stem internodes (${\mathrm{i}}_{1}$, ${\mathrm{i}}_{2}$, ${\mathrm{i}}_{3}$, and ${\mathrm{i}}_{4}$ from the tip) during the gravitropic response was measured by tracking cauline leaves using time-lapse images (Fig. 2f, left, Figs. S3 and S4). The relative elongation ratio of each stem internode was evaluated to be approximately 1.0, even for the most bent internodes (Fig. 2f, right), implying that stem bending was achieved despite the weak axial elongation.

### A mathematical model explores the stretching and bending forces during hyperbending

**Figure 3:**
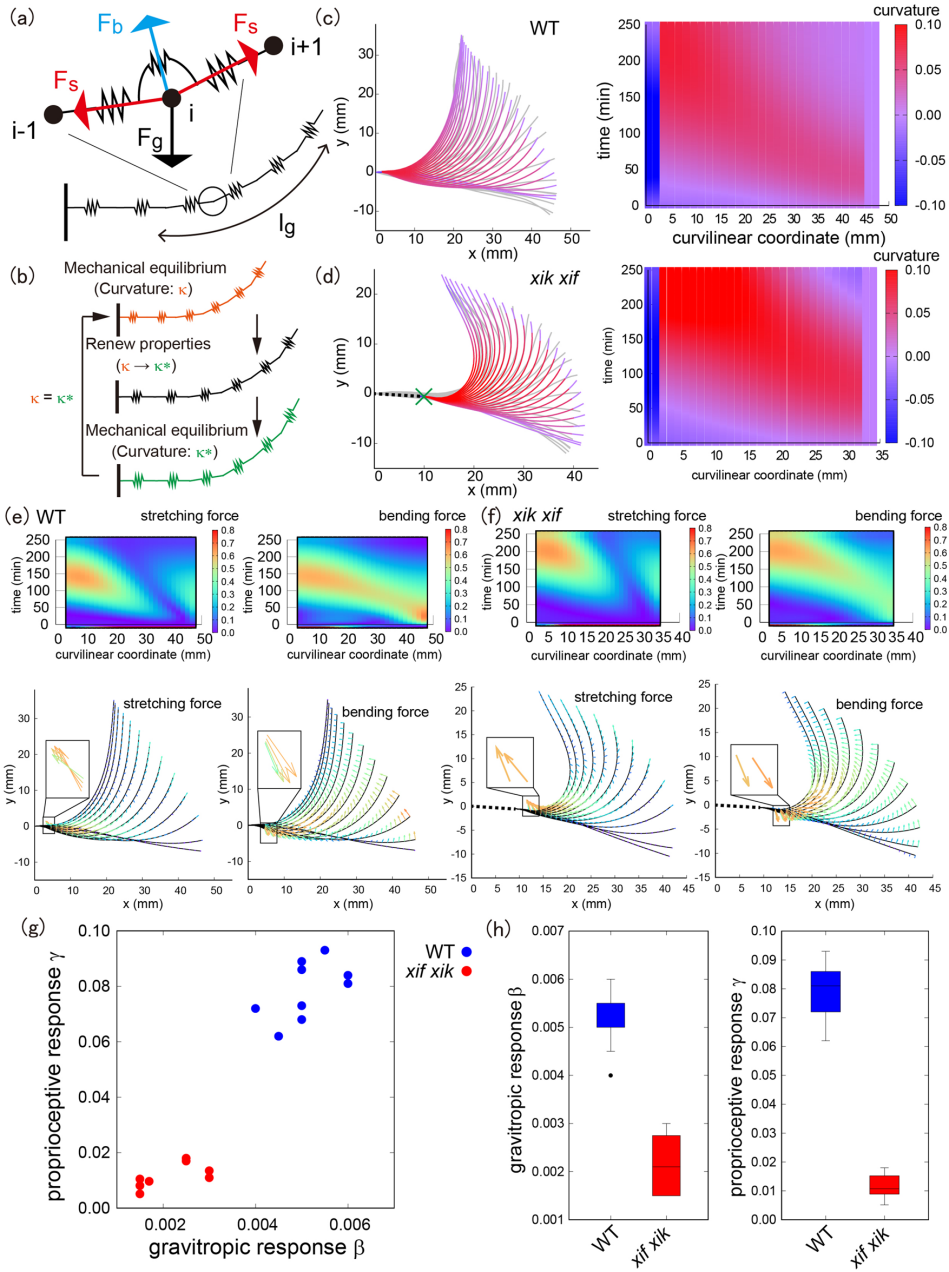
Extraction of mechanical forces during hyperbending and meaningful model parameters. (**a**) Schematic illustration of the mechanical model. (**b**) Calculation process of stem dynamics. After mechanical equilibrium at the current time, the intrinsic curvature along the stem is renewed and reaches the next mechanical equilibrium. (**c**,**d**) A representative stem bending with colored spatio-temporal curvature on top of actual stem data (gray) for wild type (**c**) and for *myosin xif xik* (**d**). The green cross mark denotes the supporting point mentioned in the main text. Right panels show the color plots of the curvature as a function of time (min) and curvilinear coordinate (mm). (**e**,**f**) Extracted stretching and bending forces for wild type (**e**) and for *myosin xif xik* (**f**). (**g**) Plot of gravitropic response *β* and proprioceptive response *γ*. (**h**) Boxplots of *β* and *γ*.

The spatio-temporal characteristics of stem bending without growth along the axial direction (Figs. 1, 2) prompted us to investigate the earlier mathematical models^11–14^. We implemented an active elastic rod model under gravity while ignoring axial growth (see “Methods”). In this model, the stem shape was determined by the intrinsic (or spontaneous) curvature of the stem segments as a function of time and space. At each time point, the stem shape was determined by balancing the momentum and force of the rod under intrinsic curvature. To compute the stem shape, we discretized the stem centerline into segments of elastic springs (see also Refs.^12,13^). The positions of the vertices (spring endpoints) are determined by the balance of the stretching force ${\mathrm{F}}_{\mathrm{s}}$ (derived from axial growth), bending force ${\mathrm{F}}_{\mathrm{b}}$ (derived from differential growth), and gravitational force ${\mathrm{F}}_{\mathrm{g}}$ (Fig. 3a). The meanings of the stretching and bending forces are detailed in the “Methods” section. We repeated the update of the intrinsic curvature and current configuration to compute the stem shape over time (Fig. 3b). It should be noted that the basal end of the model was fixed at a specific point, the supporting point, where the basal side beyond the supporting point did not change significantly with time (Fig. 3d). The stiffness of the elastic rod for wild type and *myosin xif xik* was assumed to be the same because the deformation rate of the *myosin xif xik* stem was not significantly different from that of the wild-type stem in a compression assay^9^.

According to the experimental data, we attempted to narrow the range of the model parameters and finally considered two parameters (gravitropic response $\beta$ and proprioceptive response $\gamma$) to be variable. We searched for the best-fitted set (${\beta }_{data}$ and ${\gamma }_{data}$) and reconstructed stem dynamics with similar initial and final shapes at 240 min (see “Methods”). As shown in Fig. 3c,d, the models qualitatively captured the actual stem bending at the beginning and around 240 min, although the detailed shape during bending could not be perfectly reconstructed (see Figs. S5, S6 for all examples).

Using these models, we obtained the mechanical information (stretching and bending forces), as shown in Fig. 3e,f (see Fig. S7 for other examples). More precisely, the displacement of the stem position was decomposed into components derived from the stretching force and those derived from the bending force, which is, the vectors in Fig. 3e,f represent the components of displacement. As demonstrated in our previous study^14^, the stretching force was derived from the axial change and current curvature of the stem. On the one hand, the stretching force suppresses bending near the tip owing to axial tension against the bending force but enhances bending near the supporting point owing to axial compression of the elastic rod (Fig. 3e, left and f, left). On the other hand, the bending force suppressed bending near the supporting point but enhanced bending near the tip (Fig. 3e, right and f, right). The bending force near the tip gradually weakened in the wild type, while it remained constant in the case of *myosin xif xik*, indicating that the *myosin xif xik* stem failed to straighten. In other words, the wild-type stem has a mechanical mechanism that reduces the bending force associated with the differential growth.

Next, the model parameters (gravitropic response $\upbeta$ and proprioceptive response $\upgamma$) were quantitatively obtained (Fig. 3g,h). These two parameters were lower in *myosin xif xik* than in the wild type, indicating that both gravitropic and proprioceptive responses are weak in *myosin xif xik*. This is a new aspect of posture control as revealed by our mathematical model. Although gravitropic and proprioceptive responses were almost impossible to distinguish based solely on organ shape, we could evaluate the difference through modeling. Interestingly, the modeling prediction of low gravitational response in *myosin xif xik* was supported by the results that more amyloplasts were localized at the bottom of the endodermal cells with lesser motility in the vertically-grown *myosin xik-* and *myosin xif xik*-stems than in the vertically-grown wild type stems (Fig. S9), suggesting that the saltatory movements of the amyloplast are suppressed in these mutants. Previous experiments have shown that expressing Myosin XIk:YFP promotes sedimentation of amyloplasts in *myosin xi1 xi2 xik* when the inflorescence stems were reoriented 180°^17^. Considering that no *Myosin XIf* promoter activity was detected in endodermal cells of the stem^9^, this suggests that amyloplast dynamics, in *myosin xif xik,* were suppressed mainly because of the lack of myosin XIk.

Taken together, our model showed that the wild type has a more straightened morphology than *myosin xif xik*. This, in turn, raised the question of whether this straightened morphology was indeed a mechanically stable state.

### Mechanical simulation by the finite element method reveals that stem posture in the wild type is a mechanically favorable shape

**Figure 4:**
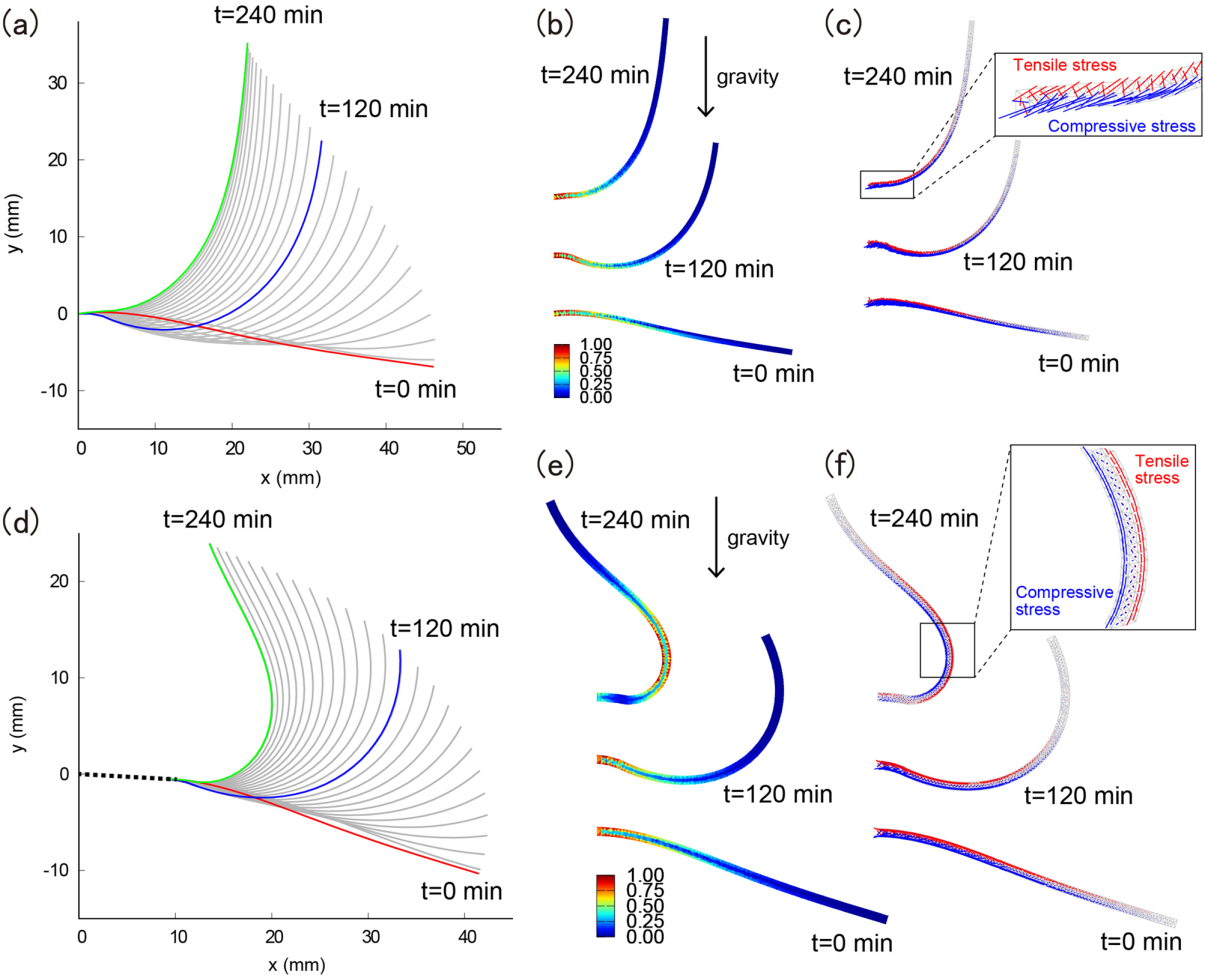
Mechanical test of the stem under a gravitational force via finite element method simulations. (**a**,**d**) Three examples of the stem shape at t = 0 (red), 120 (blue), and 240 min (green) with the same samples in Fig. 3 for wild type (**a**) and for *myosin xif xik* (**d**). (**b**,**e**) von Mises stress under the gravitational force for wild type (**b**) and for *myosin xif xik* (**e**). (**c**,**f**) Principal direction of stress with color indices of tensile stress (red) and compressive stress (blue) for wild type (**c**) and for *myosin xif xik* (**f**).

Based on the experimental data of spatio-temporal bending, we hypothesized that the posture of wild-type stems is mechanically beneficial in terms of mechanical stress due to self-weight. To test this hypothesis, we employed a finite element method simulation with morphology in the model obtained at $t=0$, $120$, and $240$ min with a slight addition of loading in the gravitational direction (Fig. 4a,d, Fig. S8). This showed that the von Mises stress under gravitational force was higher at the supporting point in the wild type; however in the mutant, the highly stressed region moves away from the supporting point at 240 min (Fig. 4b,e). More precisely, the wild-type stem experienced tensile stress at the upper flank and compressive stress at the lower flank, which is beneficial to the supporting point (Fig. 4c). On the other hand, at 240 min, the middle region of the *myosin xif xik* stem shows an inverted stress profile with tensile stress in the right flank (initially compressed flank) and compressive stress in the left flank (initially tensioned flank) (Fig. 4f). The stress in the middle is much higher than that the supporting point in the inverted stress direction, indicating that the stem shape in *myosin xif xik* is a mechanically disadvantageous posture. Therefore, we conclude that the wild-type stem is more mechanically stable than the *myosin xif xik* stem.

## Discussion

In this study, we quantitatively evaluated the spatio-temporal characteristics of wild type and *myosin xif xik* stems. For the characteristic scales of time and space associated with bending, we could observe that the relative starting position of bending was closer to the tip in the *myosin xif xik* than in the wild-type stem. This observation prompted us to consider a supporting point in the theoretical model that describes a modified growth region distributed near the tip of the *myosin xif xik* stem. Additionally, using the tip angle information, we could quantitatively capture the hyperbending behavior in the *myosin xif xik* stems. Interestingly, we found that the change in stem axial length was weak in the region near the tip over the 240 min time interval, indicating that axial growth of the stem may not be a dominant driving force during bending. This question of whether axial growth is necessary should be further confirmed experimentally, for example, by monitoring water transport in the upper and lower flanks during gravitropism.

One possible way to disentangle the mechanical forces during stem bending is to investigate the mathematical models. With modeling, we could identify when and where the mechanical forces are applied and the type of mechanical forces during bending (stretching force derived from the axial elasticity and bending force derived from differential growth), which were indistinguishable by observing the stem appearance during experiments. Our model also allowed us to determine that the duration of the applied bending force at the tip was much longer during hyperbending in *myosin xif xik*. As the cause of the bending force is differential growth, this suggests that the control mechanism of differential growth, especially in the tip region, was disrupted in *myosin xif xik* stems. In addition, in both wild-type and *myosin xif xik*, the mechanical forces at the basal region were larger than those in the other regions where the stretching and bending forces were applied in the opposite direction (Fig. 3e,f). This mechanical information predicts that biological events in the basal region of the stem might be different from those in other regions. A possible explanation for this could be cell wall stiffening in the basal region of the stem. Previous report suggests that the apical-to-basal decrease in elastic and plastic compliances along the Arabidopsis inflorescence stem is caused by pectin structural changes^18^.

Finally, we investigated whether the more straightened posture in the wild type was a mechanically optimized shape and evaluated it using finite element method simulations. To confirm the mechanics during bending, we constructed a finite-element-method-based elastic rod under gravity, which was different from our mathematical model with dynamic differential growth. We observed abnormally applied tensile and compressive stresses in the middle of the *myosin xif xik* stem, which were reduced in the wild-type stem, indicating that the posture of *myosin xif xik* was mechanically unfavorable. This could be the reason why the supporting point in the *myosin xif xik* stem shifted towards the tip, because the supporting point needed to achieve a mechanical balance at a position closer to the tip to reinforce the unbalanced shape. Thus, our results may shed light on mechanically suitable postures for various shapes, with the wild-type shape being a more mechanically suitable posture than the unbalanced posture in *myosin xif xik*. We speculate that this unbalanced shape and continuous balancing through straightening might be related to the mechanics of circumnutation^19^ and stem behavior with combined light-gravity stimulation^20^, which will be the next targets for analysis using finite element method mechanical tests.

Gravitropism consists of two processes: gravitropic organ bending and following organ straightening. In this study, we found that the gravitropic response of *myosin xif xik* was weaker than that of the wild type (Fig. 1g). Nevertheless, the inflorescence stem of *myosin xif xik* exhibited an oscillatory behavior in which they were once bent up to 150° and then returned to around 80° during gravitropic response for 8 h^9^. Considering a serious defect in the organ straightening in *myosin xif xik*, the oscillatory behavior is thought to reflect simply gravitropic bending as previously expected theoretically^11^. Although the straightening mechanism is largely unknown at present, our results from the mechanical model suggests that a trigger for the organ straightening may be tensile or compressive stress applied to cells. Since we have identified actin and myosin XI as factors required for the organ straightening, it is possible that the cytoskeleton acts as a sensor or an actuator of mechanical stress.

With the aid of spatio-temporal evaluations using (1) quantitative data analysis, (2) mechanical characterization with mathematical modeling, and (3) hypothetical static mechanical testing with the finite element method, we conclude that the straightening behavior in wild-type stems is a mechanically essential process. This type of interdisciplinary research may serve as a blueprint for reimagining interesting biological behavior to mathematical and mechanical research problems.

## Methods

### Plant materials and growth conditions

*Arabidopsis thaliana* (accession Columbia-0 [CS60000]) was used as the wild-type plant. The T-DNA-tagged mutants *myosin xif-1* (*myosin xif*)^9^, *myosin xik-2* (*myosin xik*)^21^, and *myosin xif-1 myosin xik-2* (*myosin xif xik*)^9^ have been described previously. *A. thaliana* seeds were surface-sterilized and then sown in plates of Murashige–Skoog medium containing 1% (w/v) sucrose, 0.5% (w/v) MES-KOH (pH 5.7), and 0.5% (w/v) Gellan gum (FUJIFILM Wako Pure Chemical Corporation) under continuous light at 22 °C for 2 weeks before being transferred to the soil. All methods were carried out in accordance with relevant guidelines.

### Gravitropism assay of inflorescence stems

Intact plants, 4–6 cm long primary inflorescence stems, were set horizontally in a growth chamber (NK systems) at 22 °C with non-directional dim light (0.06 μmol m^−2^ s^−1^), which is too low to induce phototropic bending of primary inflorescence stems. They were photographed automatically every 10 min using a digital camera (Canon).

### Imaging of the amyloplasts in vertically-grown plants with a vertical stage microscope

Imaging of the longitudinal section (1 cm in length, 1–2 cm from the top of the inflorescence stem of 4–8 cm height) with a vertical stage microscope was performed as described previously^22^. Differential interference contrast images were taken at 1-s intervals for 3 min. Amyloplasts in 8–13 time-lapse images (at least 10 cells) were manually tracked using the ImageJ software.

### Mathematical model

Our mathematical model is based on the growing elastic rod in the reference^12^, and the calculation process is the same as that in our previous work^14^. The centerline of the stem is modeled by a curve whose position vector at time $t$ is given by $\overrightarrow{r}\left(s,t\right)=(x\left(s,t\right),y(s,t))$ where s is the curvilinear coordinate along the stem with $0\le s\le {L}_{t}$ and ${L}_{t}$ is the total length at time $t$. The stem angle at position $s$ is defined as the angle between the local tangent and vertical axis (see Fig. 1b). The basal end of the stem is clamped with $\theta \left(0,t\right)=\pi /2$, $x\left(0,t\right)=y\left(0,t\right)=0$, and the apical end ($s={L}_{t}$) is set to be free of force and moment at any time $t$. The shape of this model is determined by balancing moment and force of the elastic rod with the local natural length and local intrinsic curvature at every time $t$ where we assumed that the speed of the mechanical relaxation is sufficiently higher than that of the movement of the stem. In this study, the local natural length $d$ is set as the initial local length. The local intrinsic curvature of stem ${\kappa }^{*} (s,t)$ is described as

$$\frac{\partial {\upkappa }^{*}(s,t)}{\partial t}=\beta \mathrm{sin}\theta (s,t)-\gamma \kappa (s,t).$$

where $\beta$ and $\gamma$ are gravitropic and proprioceptive sensitivities, respectively^11,14^. Using the internal moment $M\left(s,t\right)$ and the force acting on the position $\mathrm{s}$ ($\overrightarrow{F}(\mathrm{s},\mathrm{t})=(H\left(s,t\right),V(s,t))$), the moment and force balance equations are given by

$$\partial M/\partial s-V\mathrm{sin\theta }-H\mathrm{cos}\theta =0,$$

$$\frac{\partial H}{\partial s}=0, \frac{\partial V}{\partial s}=-\rho g,$$

using the constitutive law given by $M=B(\kappa -{\kappa }^{*})$. $B$ is the bending modulus, and $\rho$ and $g$ are the mass density per unit length and gravitational acceleration, respectively.

The force acting on the stem is composed of three components. The first is the gravitational force for all the positions which is always directed downward to the ground. As in our previous study (Tsugawa et al.^14^), the typical stretching force was estimated as ${E}_{y}\pi {\delta }^{2}$~8N with Young’s modulus ${E}_{y}\sim 10$ MPa and the radius of the stem $\delta \sim 0.5$ mm, and the typical bending force was estimated as $D/{L}_{0}^{2}\sim 3\times {10}^{-4}$ N with the bending modulus $D\sim 4.9\times {10}^{-7}$ ${\mathrm{Nm}}^{2}$ and the initial stem length ${L}_{0}\sim 40$ mm, therefore, the gravitational force $\pi {\delta }^{2}{L}_{0}\rho g/n\sim 6.0\times {10}^{-6}$ N with the mass density $\rho \sim {10}^{3}\, \text{kg/{m}}^{3}$^12^ and the discretized number of particles outlined below $n=50$ is estimated as negligibly small in the simulation. The second is the bending force corresponding to the force derived from cell activities which is related to differential growth. When the lower flank elongates more strongly than the upper flank, a bending force acts in the direction of the stem bend. When the upper flank elongates more strongly than the lower flank, the bending force acts in a direction that straightens the stem. We note that axial growth was not observed in our experiments on cauline leaves (Fig. 2), and we assumed that the length of the centerline did not change even when differential growth occurred (e.g., one side elongates and the other side shrinks). The third is the stretching force corresponding to the force derived from both the axial elastic properties of the cells and the axial growing force of the cells. The latter was not considered in this study. The former is the major stretching force in this study, where the direction is determined by the balance of the three forces. We note that it can be a stretching force that resists the bending force, or a compressive force that resists organ straightening. The stretching force is determined by assuming that the stem acts as an elastic rod that satisfies mechanical equilibrium.

To simulate the stem shape, we discretized the centerline into a set of particles connected by elastic bonds. The position of the $i$th particle was updated based on the balance equations of the force and moment described above. The elastic force at the $i$th particle was computed from the stretching of the bond ${b}_{i}=|{r}_{i+1}-{r}_{i}|$ and the angle of adjacent bonds ${\phi }_{i}$. The stretching and bending potentials are respectively given by ${U}_{s}=\left(\frac{E}{2}\right){\sum }_{i}{\left({b}_{i}-{d}_{i}\right)}^{2}$ and ${U}_{b}=\left(\frac{B}{2}\right){\sum }_{i}{\left({\phi }_{i}-{\phi }_{i}^{*}\right)}^{2}$ with the elastic parameter $E$ calculated from Young’s modulus. ${d}_{i}$ and ${\phi }_{i}^{*}$ are the natural length of the bond and the natural angle of the adjacent bonds (calculated from ${\kappa }^{*}$), respectively (see details in the reference^14^). The total force applied to the $i$th particle is given by

$${{\varvec{F}}}_{{\varvec{i}}}={{\varvec{F}}}_{{\varvec{s}}}+{{\varvec{F}}}_{{\varvec{b}}}+{{\varvec{F}}}_{{\varvec{g}}}=-{\nabla }_{{\mathrm{r}}_{\mathrm{i}}}{U}_{s}-{\nabla }_{{\mathrm{r}}_{\mathrm{i}}}{U}_{b}-{\nabla }_{{\mathrm{r}}_{\mathrm{i}}}{U}_{g},$$

where ${U}_{g}$ represents the gravitational potential. In the discretized form, the curvature equation is expressed as

$${\kappa }_{i}^{*}\left(t+\Delta t\right)={\kappa }_{i}^{*}\left(t\right)+\left(\beta \mathrm{sin}{\theta }_{i}-\gamma {\kappa }_{i}\right)\Delta t.$$

The position of the particle is updated using damped Verlet method.

### Determination of model parameters from data

To determine the best-fitted parameters of $\beta$ and $\gamma$ in the model, we calculated the deviation between the data and model $\delta$ including the deviation of the initial inclination angle at the tip ${\alpha }_{0}^{data}-{\alpha }_{0}^{model}(\beta ,\gamma )$ and that of the final inclination angle at the tip at 240 min ${\alpha }_{240}^{data}-{\alpha }_{240}^{model}(\beta ,\gamma )$ as follows:

$$\delta \left(\beta ,\gamma \right)=\sqrt{{\left({\alpha }_{0}^{data}-{\alpha }_{0}^{model}(\beta ,\gamma )\right)}^{2}+{\left({\alpha }_{240}^{data}-{\alpha }_{240}^{model}(\beta ,\gamma )\right)}^{2}}.$$

In this study, we first searched for a parameter set $(\beta ,\gamma )$ with which the angle ${\alpha }_{0}^{model}(\beta ,\gamma )$ becomes equal to the angle ${\alpha }_{0}^{data}$. We then systematically changed the set $(\beta ,\gamma )$ around the initial set and found a local minimum of $\delta \left(\beta ,\gamma \right)$. This method enables us to fit the initial and final inclination angle of the model to the data with a precision of ${10}^{-2}$ in terms of the angles in radians; however, the detailed shape during bending could not be completely reproduced.

## Supplementary Information

Supplementary Information.

## Data Availability

The datasets for this study can be found in the Supplementary information. The data that support the findings of the mathematical model for this study are available from the corresponding author, S. Tsugawa, upon reasonable request. We share the simulation code that calculates the stem morphologies which works on web browsers at github. https://satorutsugawa.github.io/linevertex.html.
