# Supplementary material for: Shoot gravitropism and organ straightening cooperate to arrive at a mechanically favorable shape in Arabidopsis
Source: Sci Rep. 2023 Jul 17;13:11165. doi: 10.1038/s41598-023-38069-x (PMC10352312; doi:10.1038/s41598-023-38069-x)
Supplement: Supplementary file 1 — Supplementary Information. [file 41598_2023_38069_MOESM1_ESM.pdf]

Supplementary material

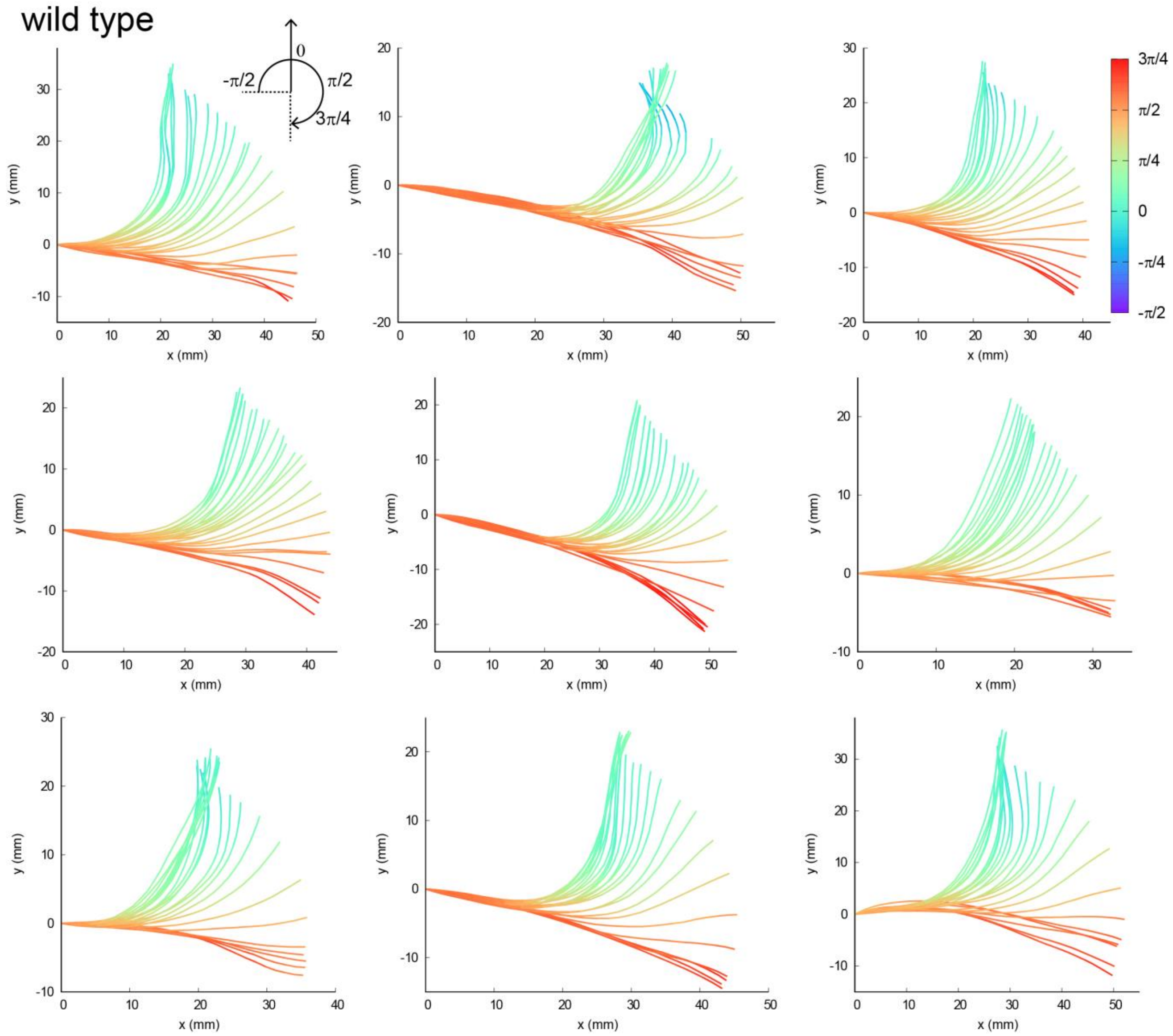

Fig. S1 | All examples of color diagram of the inclination angles of the wild-type stems.

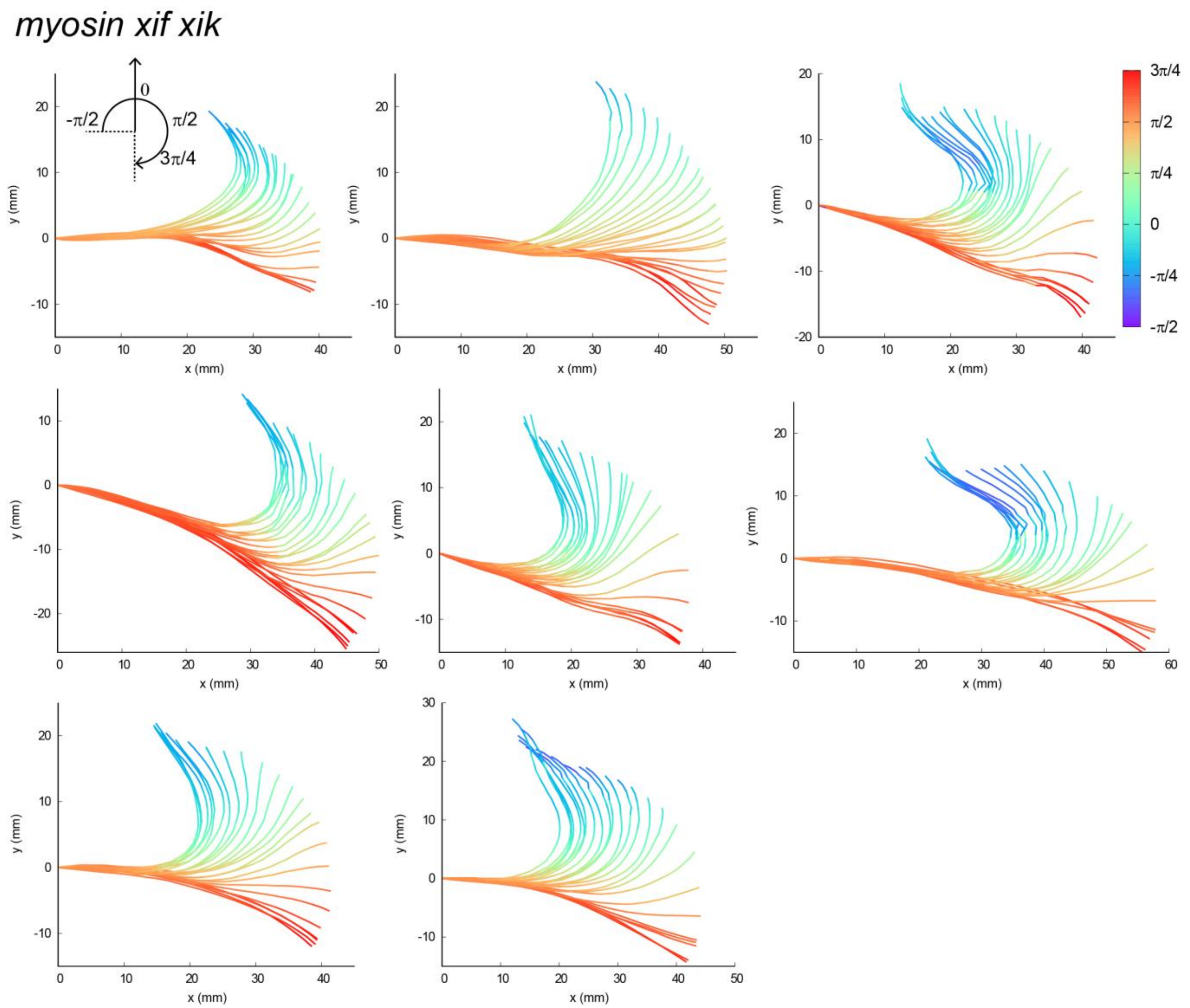

Fig. S2 | All examples of color diagram of the inclination angles of the *myosin xif xik* stems.

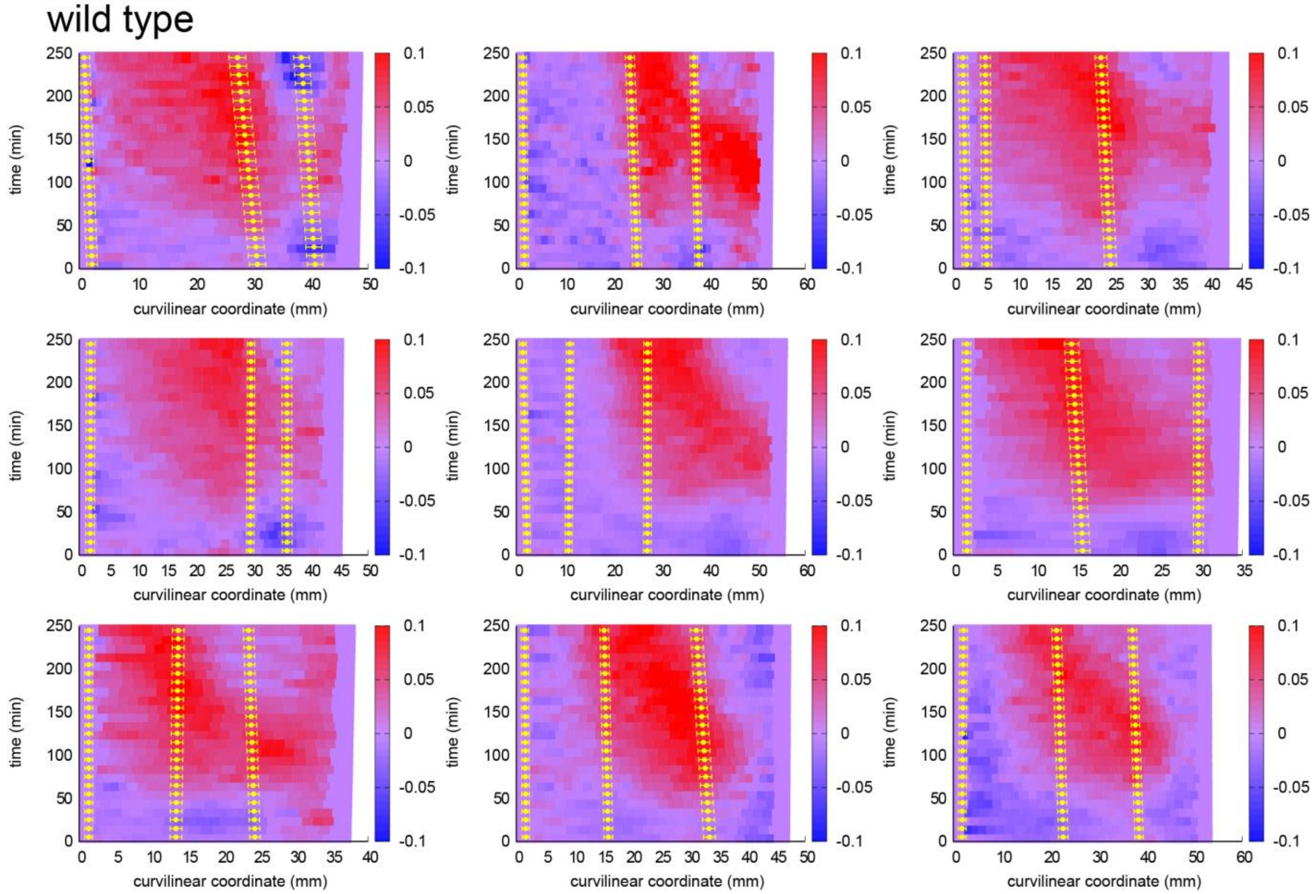

Fig. S3 | All examples of color diagram of curvatures of the stems as a function of time (min) and curvilinear coordinate (mm) in wild type. The positions of cauline leaves were also plotted.

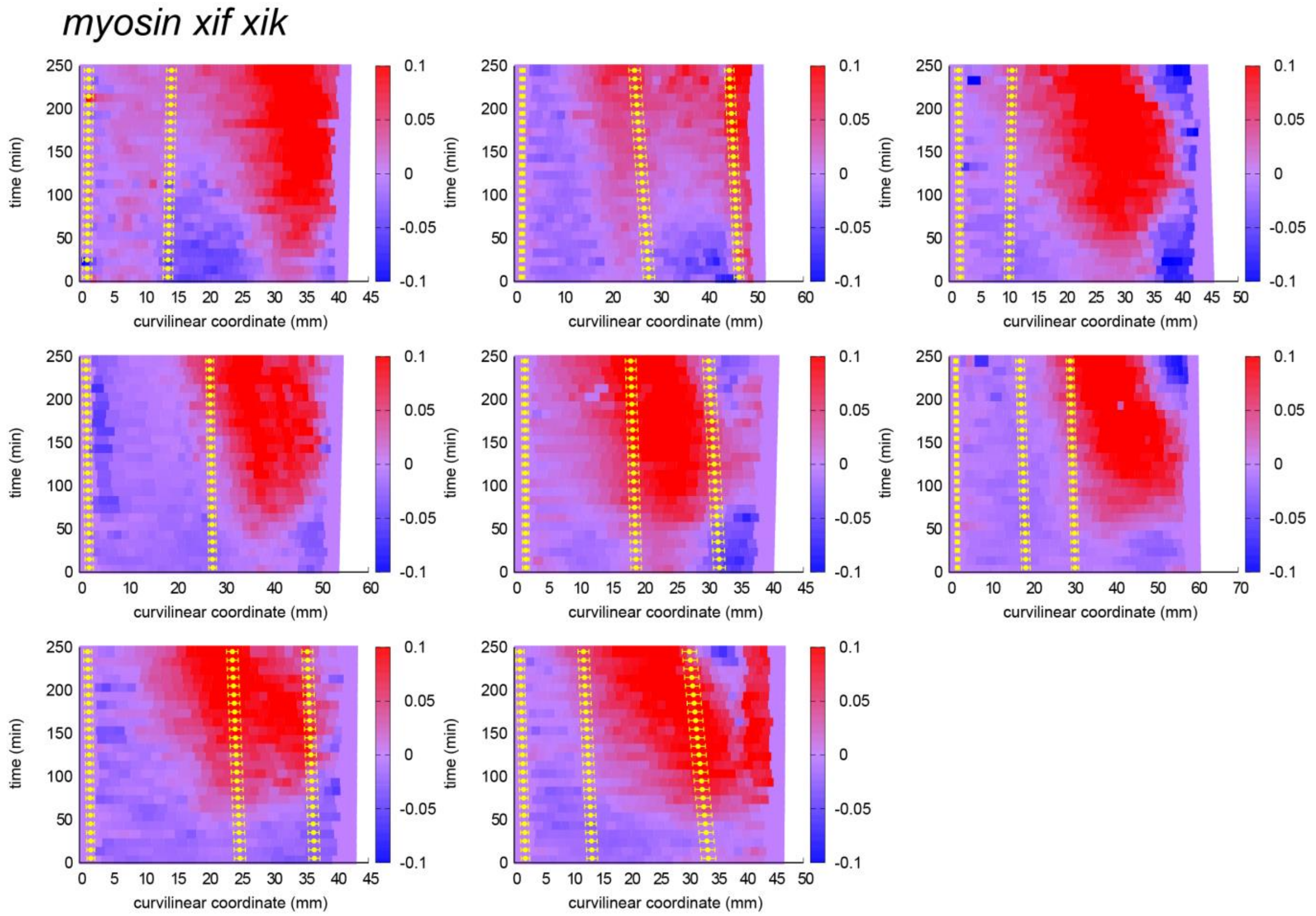

Fig. S4 | All examples of color diagram of curvatures of the stems as a function of time (min) and curvilinear coordinate (mm) in *myosin xif xik*. The positions of cauline leaves were also plotted.

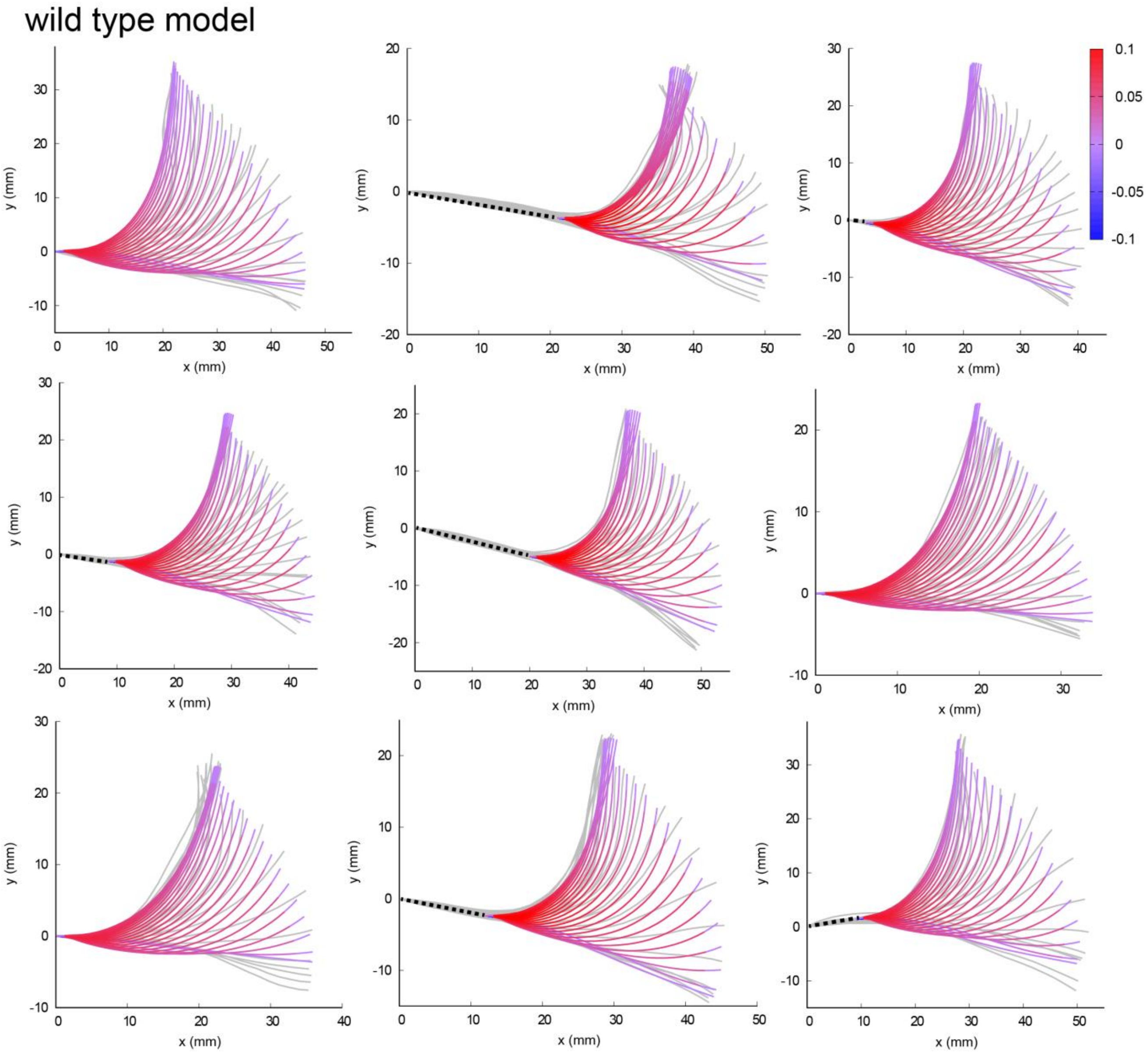

Fig. S5 | All examples of the stem bending with colored spatio-temporal curvature on top of actual shoot data (gray) for wild type.

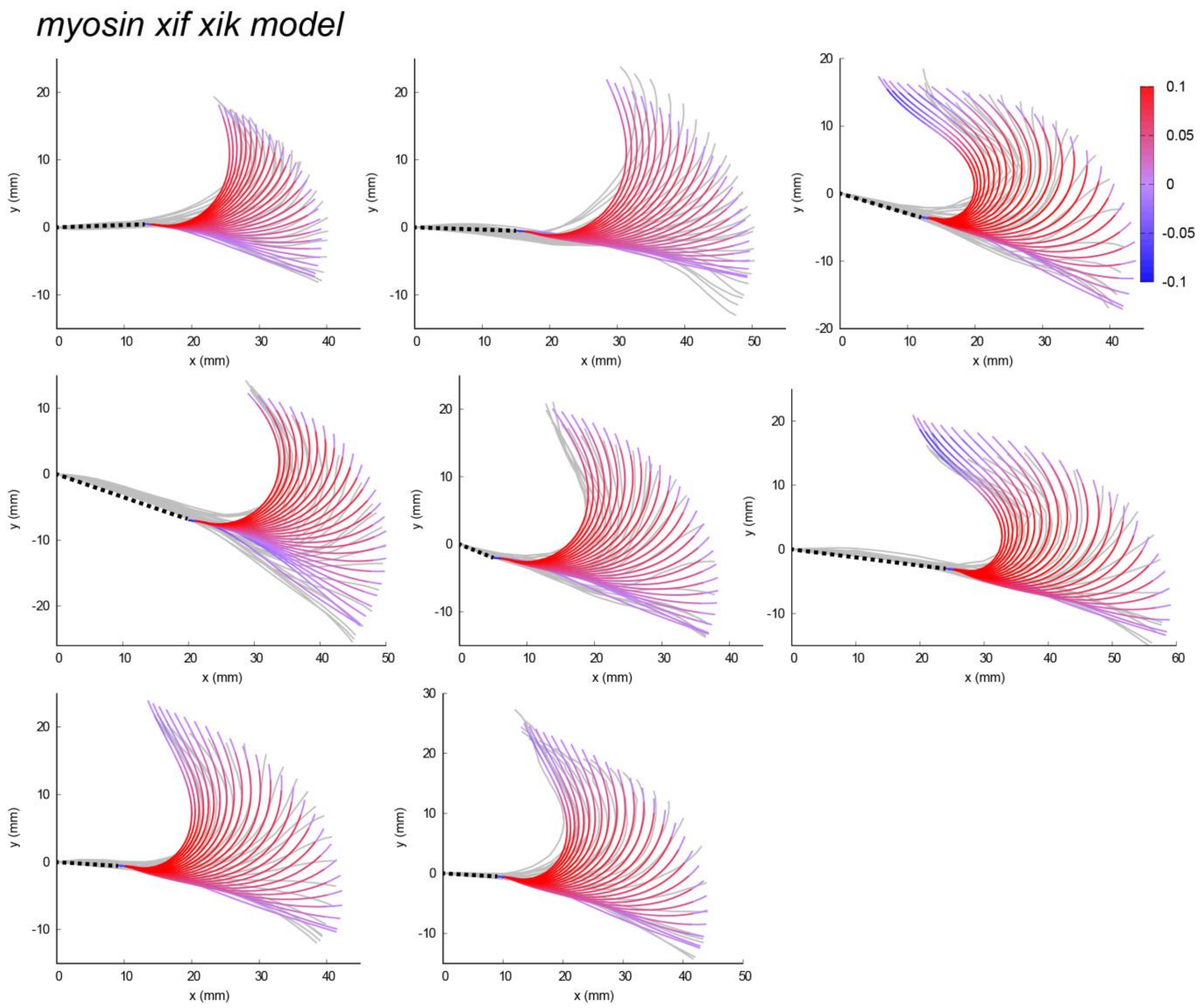

Fig. S6 | All examples of the stem bending with colored spatio-temporal curvature on top of actual shoot data (gray) for *myosin xif xik*.

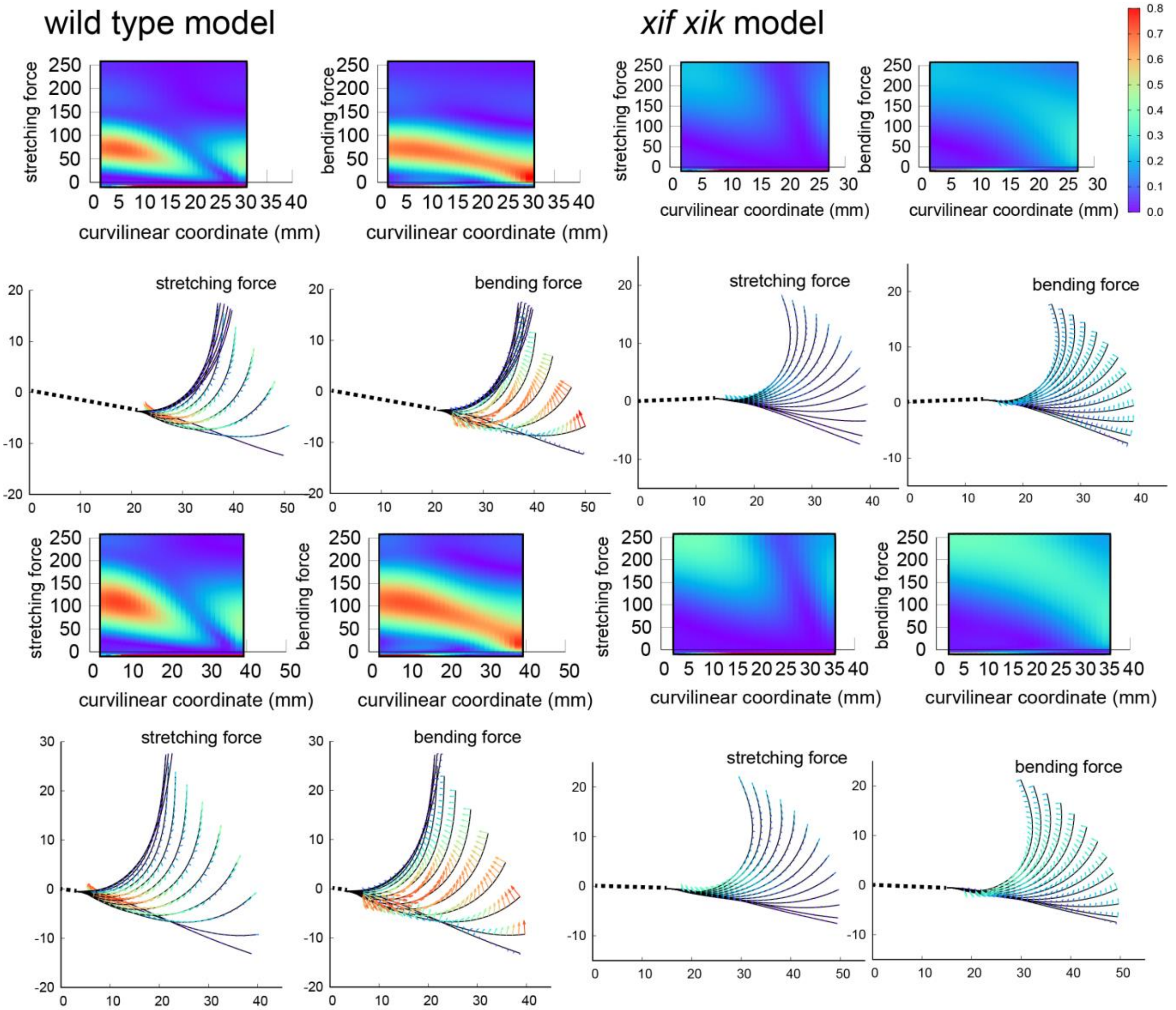

Fig. S7 | The other few examples of extracted stretching and bending forces for wild type (left four panels) and *myosin xif xik* (right four panels), respectively.

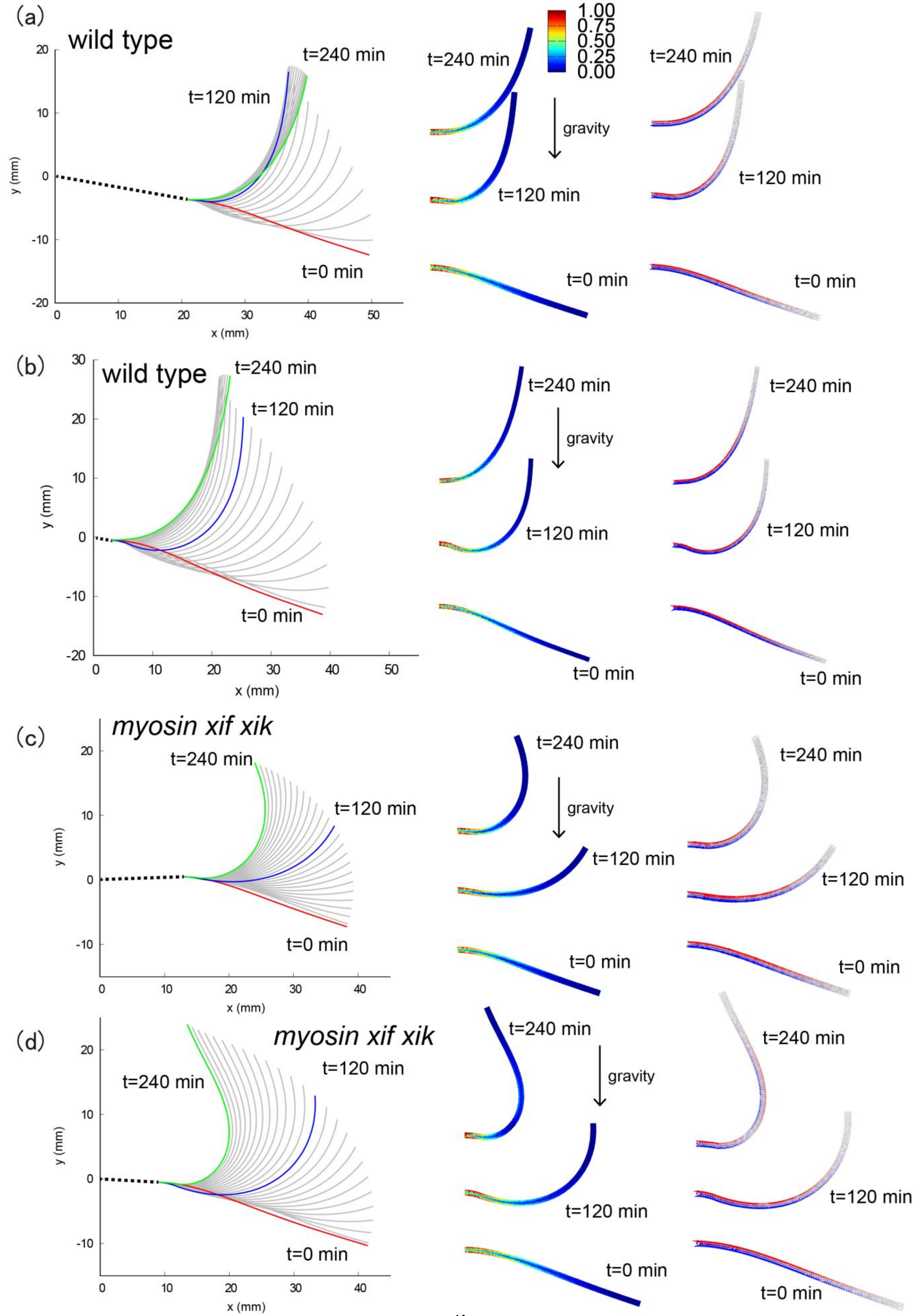

(a)
wild type
t=240 min
t=120 min
t=0 min
gravity
1.00
0.75
0.50
0.25
0.00
y (mm)
x (mm)
(b)
wild type
t=240 min
t=120 min
t=0 min
gravity
y (mm)
x (mm)
(c)
myosin xif xik
t=240 min
t=120 min
t=0 min
gravity
y (mm)
x (mm)
(d)
myosin xif xik
t=240 min
t=120 min
t=0 min
gravity
y (mm)
x (mm)

Fig. S8 | The other few examples of mechanical test adding a gravitational force with the finite element method for wild type (a, b) and *myosin xif xik* (c, d), respectively.

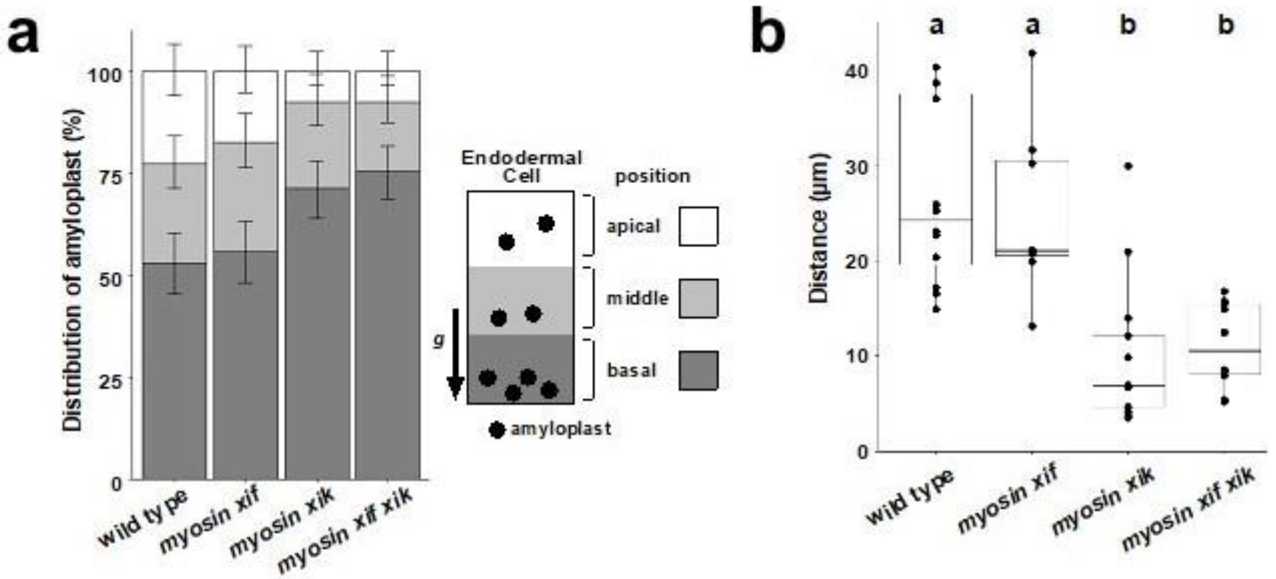

	apical : middle	apical : basal	middle : basal
WT : *xif*	0.378 n.s.	0.378 n.s.	0.953 n.s.
WT : *xik*	0.0300 *	1.56E-4 ***	0.212 n.s.
WT : *xif xik*	0.134 n.s.	1.39E-4 ***	0.0300 *
xif : *xik*	0.217 n.s.	7.69E-3 *	0.148 n.s.
xif : *xif xik*	0.516 n.s.	5.50E-3 *	0.0208 *
xik : *xif xik*	0.919 n.s.	1.00 n.s.	0.437 n.s.

Fig. S9 | Suppression of amyloplast dynamics in the endodermal cells of *myosin xik* and *myosin xif xik*. The longitudinal section of upper part of the inflorescence stem in wild type (WT), *myosin xif*, *myosin xik*, and *myosin xif xik* were observed with a vertical stage microscope. (a) Distribution of amyloplasts in the endodermal cell divided into three areas. Error bars represent binomial 95% confidence intervals. The table show the Fisher's exact test *P*-values with Benjamini-Hochberg correct. * $P < 0.05$; *** $P < 0.0005$; n.s. not significant. (b) Average distance moved by individual amyloplasts within the cell in 3 minutes. The different letters exhibit significant differences ($P < 0.05$, Tukey's method).
